# A hybrid approach for pattern recognition and interpretation in age-related false memory

**DOI:** 10.3389/fpsyg.2025.1579259

**Published:** 2025-07-23

**Authors:** Noorbakhsh Amiri Golilarz, Elias Hossain, Shahram Rahimi, Hossein Karimi

**Affiliations:** ^1^Department of Computer Science, The University of Alabama, Tuscaloosa, AL, United States; ^2^Department of Computer Science and Engineering, Mississippi State University, Starkville, MS, United States; ^3^Department of Psychology, Mississippi State University, Starkville, MS, United States

**Keywords:** aging, cognitive decline, false memory, hybrid model, large language model (LLM), behavioral patterns, modified LightGBM, similarity-based interference

## Abstract

**Introduction:**

Aging is associated with a decline in essential cognitive functions such as language processing, memory, and attention, which significantly impacts the quality of life in later years. Despite the serious consequences of age-related cognitive decline, particularly in the formation of false memories, the underlying mechanisms remain poorly understood. This knowledge gap is partly due to limitations in current methodologies used to examine age-related cognitive changes and their origins.

**Methods:**

In the present study, a hybrid approach was developed that combines optimized machine learning techniques with large-scale transformer-based language models to identify behavioral patterns distinguishing true from false memories in both younger and older adults. The best-performing model, a modified version of the Light Gradient Boosting Machine (LightGBM), identified nine key features using permutation importance. Feature interactions with age were further examined to understand their relationship with cognitive decline. Additionally, the modified LightGBM was integrated with a language model to enhance interpretability.

**Results:**

The findings revealed that younger adults benefited from target encoding time during reading, which helped them correctly reject misleading information (lures), whereas older adults were more vulnerable to inference caused by semantic similarity.

**Discussion:**

These results offer important insights into the mechanisms of false memory in aging populations and demonstrate the utility of hybrid computational methods in uncovering behavioral patterns related to memory decline. The modified LightGBM achieved the highest overall performance with an F1-score of 0.82 and recall of 0.88, outperforming all evaluated deep learning and transformer-based models.

## 1 Introduction

Memory is one of the key cognitive functions that affects the performance and participation of older adults in daily activities, as well as their personal and social life in general. Age-related decline in memory has a profound negative impact on older adults’ quality of life, by reducing their autonomy and their ability to establish and/or maintain strong social bonds, which may lead to social isolation. Importantly, social isolation may, in turn, lead to mental health issues and reduce overall well-being. Unfortunately, age-related decline in memory function is a common cognitive problem, affecting approximately 11.1% of the population in the United States (Centers for Disease Control and Prevention, 2019). Since memory loss is indeed a frustrating and life-changing cognitive problem, it is important to empirically investigate the associated underlying mechanisms to facilitate the development of effective diagnostic methods and treatments.

Moreover, a particular aspect of memory deterioration is the increased susceptibility of older adults to false memories—where a memory trace associated with an event is erroneously recalled or recognized as having occurred. The Inhibition Deficit Hypothesis (IDH) offers a widely accepted theoretical explanation, suggesting that aging impairs the ability to suppress irrelevant information during memory retrieval, thereby increasing cognitive interference. However, while such theory-driven frameworks have provided valuable insights, they typically rely on isolated hypothesis testing and focus only on predefined variables. The conventional approach may fail to capture important features or interactions that are not predicted by theory and may neglect complex and latent patterns in behavioral data. Consequently, reproducibility and generalizability frequently deteriorate, particularly when research conditions differ between studies (Open Science Collaboration, 2015; Johnson et al., 2017; Nosek et al., 2022). This exposes a critical gap: the absence of data-driven, scalable methodologies that can strengthen current theories and disclose concealed cognitive and behavioral patterns that contribute to memory decline and false memory susceptibility.

In light of this, the current study investigates the application of modern computational techniques—specifically, machine learning (ML) and large language models (LLMs)—to analyze a dataset on age-related differences in true and false memories. ML provides powerful tools for uncovering non-obvious relationships in high-dimensional data (Lv et al., 2020; Naghshvarianjahromi et al., 2019), while LLMs enhance interpretability by offering natural language-based explanations of results. Together, these technologies offer a robust, hybrid framework for identifying key predictors and cognitive-behavioral patterns associated with memory performance across age groups (Bhatt et al., 2023; Nematollahzadeh et al., 2020; Wan et al., 2018).

**Figure 1:**
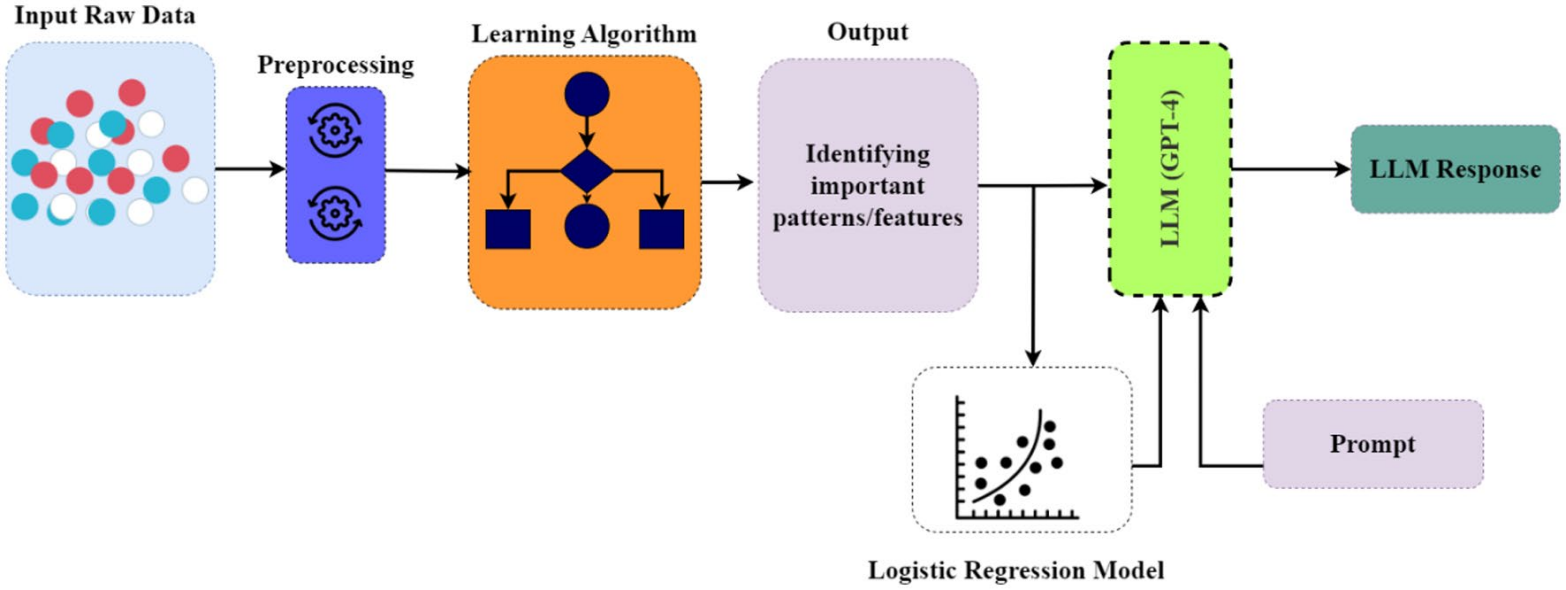
Visualization of the learning algorithm training procedures and integration with the LLM.

Motivated by these challenges and opportunities, the present work explores how customized ML models, combined with semantic modeling and LLM-based prompt engineering, can enhance our understanding of cognitive aging and provide a scalable benchmark for future research in memory and aging. The key contributions of this study are as follows. We:

- Customized existing machine learning methods to delve deeper into the dataset and identify significant regularities and factors contributing to memory performance and susceptibility to false memories.
- Modified the LightGBM model to capture semantic relationships within the dataset for identifying cognitive decline and false memory patterns, providing a benchmark for the research community.
- Integrated the LLM prompt engineering technique with the modified LightGBM model to generate consistent and conceptually informative responses for understanding false and true memory patterns across different age groups.
- Conducted rigorous feature interaction modeling to determine the most important factors directly related to the underlying cognitive phenomena (see Figure 1).

The remainder of this paper is structured into four sections. Section 2 presents the prior research on cognitive aging and memory modeling. Section 3 outlines the research methods, including the machine learning models implemented and the strategies used for their enhancement through hyperparameter optimization. Section 4 reports the findings, accompanied by detailed analyses and insights. Finally, Section 5 offers the main conclusions of the manuscript.

## 2 Prior research

Previous research has demonstrated that older adults are more susceptible to false memories than younger adults (Devitt and Schacter, 2016; Abichou et al., 2021). False memories occur when a memory trace associated with an event is erroneously recalled and/or recognized as having occurred in the past. An influential theory that provides an explanation for false memory in older adults is the Inhibition Deficit Hypothesis (IDH) (Zacks and Hasher, 1988; Lustig et al., 2007). According to this theory, aging leads to a decline in the ability to inhibit information that is distracting or irrelevant during memory retrieval. Importantly, availability of such irrelevant information increases interference during the retrieval of target memory items, and if this interference is strong enough, the activation level of non-target (irrelevant) memory items may reach the recognition threshold, creating false memories for those items.

In other words, inability to suppress irrelevant information may reduce the discriminability of stored memory items, leading to false recognition and/or recall. Empirical evidence for the IDH comes from studies showing that older adults’ performance declines in tasks requiring the suppression of irrelevant information (May and Hasher, 1998; Andrés et al., 2006), as well as studies showing that older adults exhibit more intrusions from irrelevant information during the retrieval of previously encoded information. In addition, this theory receives support from neuroimaging studies showing that, relative to younger adults, older adults have reduced activation in brain regions associated with inhibitory control (e.g., the prefrontal cortex; Gazzaley et al., 2005), when task performance relies on cognitive inhibition.

In a different domain, Antonijevic et al. (2025) introduced a hybrid AI framework to improve the security of IoT devices integrated with the Metaverse. By combining CNN with machine learning models such as CatBoost and LightGBM, and optimizing them using metaheuristics, the framework effectively identified and classified intrusions in IoT networks. Their two-level architecture achieved multi-class classification accuracy of up to 99.83% in a real-world dataset. Additionally, explainable AI was incorporated to interpret model decisions and support future improvements.

Similarly, Villoth et al. (2025) proposed a dual-layer AI framework for software fault detection using a combination of natural language processing and machine learning. A CNN was employed for feature extraction, while boosting classifiers such as XGBoost, AdaBoost, and CatBoost were used in the second layer, all tuned via a modified firefly algorithm. With custom TF-IDF encoding, their model achieved up to 99.8% accuracy across seven experiments using public datasets.

Likewise, Dobrojevic et al. (2024) addressed sexual harassment detection on Twitter through natural language processing and machine learning. They used TF-IDF and BERT for feature representation and employed XGBoost models optimized with a modified Coyote Optimization Algorithm. SHAP was applied to interpret model behavior, revealing insights into the patterns of online harassers.

While these studies illustrate the efficacy of hybrid deep learning and machine learning methodologies across diverse fields—such as IoT security, software testing, and online abuse detection—they do not explicitly investigate their relevance to age-related cognitive decline or false memory prediction. The current literature is deficient in data-driven frameworks that combine structured feature modelling with interpretability tools to elucidate the underlying patterns of memory failures in older individuals. Our proposed method solves this deficiency by utilizing machine learning to reveal the behavioral and cognitive patterns that contribute to false memory susceptibility in ageing populations.

## 3 Methods

To better analyze and capture insights in the dataset, several machine learning (ML) algorithms, deep learning (DL) models, and cutting-edge transformer models were employed. The modified ML models demonstrated more satisfactory performance compared to traditional DL or transformer-based approaches. As the objective was to uncover hidden patterns within the dataset, ML models were customized to maximize their efficiency. This customization was necessary because typical ML models function as black boxes, where internal parameter operations are not easily interpretable during runtime. These models are generally pre-configured for various downstream tasks in real-world scenarios. Therefore, existing models were modified to enable more appropriate extraction of information and patterns. To further enhance model efficiency and ensure generalization, hyperparameter configurations were performed using grid search cross-validation techniques, and regularization methods such as L1 (lasso) and L2 (ridge) were applied to mitigate overfitting issues. These steps supported the development of a more robust and unbiased model. In the following subsections, various cutting-edge machine learning models and their optimization settings are discussed, along with the hyperparameter tuning strategies employed in this study.

### 3.1 Decision tree

The decision tree algorithm has two main types: Classification and Regression Trees (CART) which handle both classification and regression tasks, and the Iterative Dichotomiser 3 (ID3) algorithm which is used specifically for classification tasks (Lu et al., 2022). These trees were constructed using a top-down methodology which implies that the tree’s root node always stays at the top of the structure and that the tree’s leaves represent the outcomes. These two varieties of trees differ significantly from one another. In contrast to CART, which performs better with continuous variables and is thus referred to as regression, the ID3 effectively classifies variables. Regarding the metrics, CART applies the Gini index, and ID3 applies the Information Gain. To further emphasize, the Information Gain is one way to quantify purity. The tree may produce more pure nodes if the information gain is higher. Conversely, the Gini index quantifies the purity of a node and goes to 0 when every member belongs to the same class. We used Scikit-Learn package (Pedregosa et al., 2011), which is based on CART. Thus, we explain its mathematical justification in the following.

If we assume that there are C classes and that $P_{c}$ is the probability that an instance belongs to the $c^{\mathrm{th}}$ class [17], then the probability distribution of the Gini index can be represented as shown in Equation 1:


(1) $$\text{Gini}(q)=1-\sum_{c=1}^{C}q_{c}^{2}\;$$

where,

-
Gini(q) represents the Gini index for a specific probability distribution *q* across the classes in a node.
-
$\sum_{c=1}^{C}q_{c}^{2}$ indicates the total squared probability of all classes c , where c represents each distinct class.
-
$1-\sum_{c=1}^{C}q_{c}^{2}$ reflects the impurity, where a lower value corresponds to a better decision tree split.

We optimized the model by adjusting hyperparameters such as “*maximum depth*,” “*minimum samples to split*,” and “*minimum samples per leaf*.” the “*maximum depth*” parameter regulates the trees’ maximum depth to mitigate overfitting issues. The “*minimum samples to split*” parameter ensures that a node will only be split if it contains a minimum number of samples, thereby improving model generalization. Similarly, the “*minimum samples per leaf*” parameter ensures that splits only take place when they enhance the performance of the model. However, the following values are applied in the parameters grid of the model: “*maximum depth*: *[10, 20, 30]*,” “*minimum samples to split*: *[2, 5, 10]*,” and “*minimum samples per leaf*: *[1, 2, 4].”*

### 3.2 Random Forest

Random Forest is an ensemble learning method in machine learning that uses the predictions of many trees to create a classification (Breiman, 2001). This model employs a technique known as bagging, where a subset of the original data is produced by replacement procedures and random sampling. The next step involves selecting a random collection of features for splitting at each node in the tree (Breiman, 2001). It then starts building decision trees on randomly selected features. After generating a large number of trees, they vote for the most popular class (Breiman, 2001).

Given an ensemble of classifiers $h_{1}(z),h_{2}(z),\ldots ,h_{M}(z)$ , and with the training set randomly drawn from the distribution of the random vector X,Y , the margin function can be defined by Equation 2:


(2) $$\mathrm{mg}(X,Y)={\mathrm{avg}}_{m}\;I(h_{m}(X)=Y)-\max_{c\neq Y}\;{\mathrm{avg}}_{m}\;I(h_{m}(X)=c).$$

where,

-
mg(X,Y) denotes the model’s margin on a specific input. A higher margin value corresponds to more confident predictions.
-
X represents the input feature vectors from the data samples.
-
Y is the target label corresponding to the input X .
-
$h_{m}(X)\;$ reflects the output of the m -th decision tree within the ensemble.
- *I(.)* is the indicator function that returns 1 if the condition is true, and 0 if false.
-
${\mathrm{avg}}_{m}\;$ is the average over all trees m in the forest.
-
c≠Y denotes the class that is predicted but does not match the actual class Y .

For hyperparameter configuration, different values were tested: “*maximum depth*: *[none, 10, 20, 30]*,” “*number of estimators*: *[100, 200, 300]*,” “*minimum samples to split*: *[2, 5, 10]*,” *and “minimum samples per leaf*: *[1, 2, 4]*.” This tuning helps us understand the performance and effectiveness of the model.

### 3.3 Adaptive Boosting (AdaBoost)

Adaptive Boosting (AdaBoost) is a supervised machine learning algorithm that combines multiple weak learners (who make mistakes while predicting) and makes a strong learner (who can correctly predict the target) (Freund and Schapire, 1997). It should be noted that this method finds the optimal solution with the least error in the training data samples. This training set of data can be represented by a distribution known as R . Here, R focuses on the likelihood of detecting distinct input values from the training data, while distribution *Q* reflects the likelihood of observing both input and output values. Usually, this distribution is uniformly set up such that $R(j)=\frac{1}{N}$ . In this case, R(j) denotes the probability of selecting the j -th attribute from the dataset and N is the total number of features.

Throughout the training process, the algorithm tracks a set of weights $w^{t}$ . By normalizing these weights $w^{t}$ , a distribution $q^{t}$ is calculated at iteration t . Then a weak learner receives this distribution and uses it to produce a hypothesis $g_{t}$ that has minimal error relative to the distribution. The boosting procedure updates the weight vector, $w^{t+1}$ , using the new hypothesis $g_{t}$ , and the cycle repeats. After T iterations, the final hypothesis, $g_{f}$ , is produced. The hypothesis $g_{f}$ aggregates the results of the T weak hypotheses using a weighted majority vote. The final hypothesis can be defined by Equation 3:


(3) $$g_{f}(x)=\{\begin{array}{ll} 1 & \text{if}\;\sum_{k=1}^{T}(\log\frac{1}{\gamma_{k}})g_{t}(z_{k})\geq \sum_{k=1}^{T}\log\frac{1}{\gamma_{k}},\\ 0 & \text{otherwise}. \end{array}$$

where,

-
$g_{f}(x)$ denotes the final hypothesis.
-
$\sum_{k=1}^{T}(\log\frac{1}{\gamma_{k}})g_{t}(z_{k})\;$ is the weighted sum of predictions for all weak learners.
-
$\log\frac{1}{\gamma_{k}}\;$ represents the logarithmic adjustment to the weights for the k -th observation.
-
$g_{t}(z_{k})$ is the prediction result of the t -th weak learner for the k -th observation.
- Value of 1 indicates the positive class, and value of 0 indicates the negative class.

However, to further improve the efficiency and configure the hyperparameters, the following values were used in the parameters grid of the model: *“number of estimators”: [50, 100, 200], “learning rate”: [0.01, 0.1, 0.5, 1.0]*.

### 3.4 Gradient boosting

The gradient boosting algorithm is an ensemble method because it builds a robust predictive model by adding individual additive components to its pipeline (Friedman, 2001). We can denote them as regression trees. This algorithm uses the concept of function estimation to find the optimal line where each data point closely matches. The initial step in gradient boosting is to build a base model to predict the observations in the training dataset. It can be determined by taking an average of the target columns (Friedman, 2001), and mathematically it can be defined by Equation 4:


(4) $$H_{0}(z)=\arg\min_{\beta }\sum_{j=1}^{M}L(t_{j},\beta )\;$$

where,

-
$H_{0}(z)$ indicates the initial prediction with respect to the input z.
-
$\arg\min_{\beta }\;$ denotes the importance of finding the predicted value β that reduces the overall loss function.
-
$\Sigma_{j=1}^{M}$ is the sum of all data points, i.e., 1 to M .
-
$L(t_{j},\beta )$ is the loss function that separates the actual or true value and the predicted value.

However, to enhance the performance of this model, the hyperparameters were configured using the following values: “*number of estimators: [100, 200, 300],*” “*learning rate: [0.01, 0.1, 0.2],*” and “*maximum depth: [3, 5, 7]*.”

### 3.5 Extreme gradient boosting (XGBoost)

XGBoost is a machine learning algorithm, introduced by Chen and Guestrin (2016) which uses a new tree learning method for sparse data, optimizing the loss function with gradient descent and incorporating regularization to address overfitting. Assuming ${\tilde{y}}_{k}(u)$ represents the anticipated k -th instance in the u -th iteration, we must add $g_{u}\;$ in order to minimize the following objective function defined in Equation 5:


(5) $$Q^{\left(u\right)}=\sum_{k=1}^{m}h(t_{k},{\tilde{y}}_{k}^{\left(u-1\right)}+g_{u}(z_{k}))+\Psi (g_{u})\;$$

where,

-
$Q^{\left(u\right)}\;$ represents the loss function at iteration u . For every instance k , it evaluates the model’s performance in predicting the desired outcome $t_{k}$ .
-
${\tilde{y}}_{k}^{\left(u-1\right)}\;$ illustrates the expected outcome for instance k at iteration u−1 .
-
$g_{u}\;$ highlights the prediction result for a new tree for the instance k .
-
$h(t_{k},{\tilde{y}}_{k}^{\left(u-1\right)}+g_{u}(z_{k}))$ expression indicates the loss function for every instance. It provides the difference between the true value $t_{k}\;$ and the predicted value ${\tilde{y}}_{k}^{\left(u-1\right)}+g_{u}(z_{k}))$ .
-
$\Psi (g_{u})$ denotes the regularization term, which helps in preventing overfitting in the model.

Turning into the parameters configuration to enhance efficiency of this model, the hyperparameters were optimized and experimented with different values for *“subsample rate”: [0.6, 0.8, 1.0], “maximum depth”: [3, 5, 7], “number of estimators”: [100, 200, 300], and learning rate: [0.01, 0.1, 0.2]*.

### 3.6 Categorical Boosting (CatBoost)

A gradient boosting technique that excels at handling categorical features is called Categorical Boosting (CatBoost). The training dataset is generated randomly in CatBoost (Dorogush et al., 2018). It uses several permutations to boost the algorithm’s efficiency by sampling a random permutation and obtaining gradients. These permutations are identical to those that are employed in the statistical computation of categorical attributes. Then, it employs the permutations to train discrete models and prevent overfitting issues (Dorogush et al., 2018). It trains n distinct models $M_{i}$ for every permutation σ . This implies that in order to construct a single tree, we must store and recalculate $O(n^{2})$ for every permutation σ because a tree’s building time may rise quadratically with the amount of training samples, making it inefficient for bigger datasets. Thus, it requires to improve each model $M_{i}$ by updating $M_{i}(X_{1}),\ldots ,M_{i}(X_{i})$ . As such, $O(n^{2})\;$ is regarded as the final operation.

To improve the efficiency and performance of the model, several parameters were optimized. More specifically, the following values were tested: *“tree depth: [6, 8, 10],” “learning rate: [0.01, 0.1, 0.2]”* and *“number of iterations: [100, 200, 300].”*

### 3.7 Support Vector Machine (SVM)

Support Vector Machine (SVM) aims to find the optimal hyperplane to separate data into two distinct classes (Boswell, 2002). Given k training examples $\left(z_{i},t_{i}\right)$ , where $z_{i}\in {\mathbb{R}}^{n}$ and $t_{i}\in \{-1,1\}$ , SVM seeks to maximize the margin between the two classes by finding a hyperplane defined as v·z+c=0 . The objective is to minimize ||v|| under the constraint $t_{i}(v\cdot z_{i}+c)\geq 1$ , which ensures the correct classification of the data. This can be solved using the Lagrange multiplier method, leading to the following optimization problem, as shown in Equation 6:


(6) $$\begin{array}{rr} \mathrm{minimize}:G(\beta ) & =-\sum_{i=1}^{k}\beta_{i}+\frac{1}{2}\sum_{i=1}^{k}\sum_{j=1}^{k}t_{i}t_{j}\beta_{i}\beta_{j}(z_{i}\cdot z_{j})\\ & \text{subject to}:\\ & \sum_{i=1}^{k}t_{i}\beta_{i}=0\\ & 0\leq \beta_{i}\leq D\;\;(\forall i) \end{array}$$

where, the parameter D controls the trade-off between maximizing the margin and minimizing classification errors.

### 3.8 Naïve Bayes algorithm

This algorithm is based on Bayes theorem (Huang and Li, 2011). In simple terms, the Bayes theorem calculates the likelihood of another event (Y) occurring given that one event (X) has occurred. Bayesian classification predicts the category of a new feature $v=b_{1},,,b_{2},,,\ldots ,,,b_{m}\;$ based on its properties (Huang and Li, 2011). To determine the most likely outcome, denoted as $W_{\text{Best}}$ , we calculate the likelihood of each possible category using the attributes of the instance. Mathematically, this can be expressed as shown in Equation 7:


(7) $$W_{\text{Best}}=\text{argmax}P(D_{k}|b_{1},b_{2},\ldots ,b_{m})\;$$

Using Bayes theorem, the expression in Equation 7 can be rewritten, resulting in Equation 8:


(8) $$\begin{array}{rr} W_{\text{Best}} & =\text{argmax}\frac{P(b_{1},b_{2},\ldots ,b_{m}|D_{k})P(D_{k})}{\left(b_{1},b_{2},\ldots ,b_{m}\right)}\;\\ & =\text{argmax}P(b_{1},b_{2},\ldots ,b_{m}|D_{k})P(D_{k}) \end{array}$$

where,

-
$W_{\text{Best}}\;$ represents the most probable class label.
-
argmax refers to the value of a variable that maximizes the function.
-
$P(b_{1},b_{2},\ldots ,b_{m})$ is the prior probability of the attributes $\left(b_{1},b_{2},\ldots ,b_{m}\right)$ .

However, the Naive Bayes classifier assumes that the attribute values are conditionally independent, given the target value.

### 3.9 K-nearest neighbours (KNN)

This is a classification algorithm (Cunningham and Delany, 2020) in which closest neighbors k are used to determine the choice for each data point. Consider the number of k to 5 (five neighbors) for a data point p . Assume that two neighbors belong to class S and the other three belong to V . Since most of the neighbors belong to V , it will be categorized as class V . This categorization can be carried out by simple majority voting or distance-weighted voting.

Regarding the model’s optimization, the required parameters were adjusted: number of neighbors: [3, 5, 7, 9], *“weighting method”: [‘uniform’, ‘distance’]* and *“distance metric”: [‘euclidean’, ‘manhattan’]*.

### 3.10 The voting algorithm

The Bagging algorithm (bootstrap aggregating) was introduced in 1996 (Breiman, 1996) as a technique that combines multiple classifiers generated from various bootstrap samples of the training data. A bootstrap sample is formed by randomly selecting m instances from the training dataset, allowing some instances to be selected more than once (Breiman, 1996). Given a dataset of pairs $\left\{(Y_{n},X_{n}),n=1,\ldots ,N\right\}$ , where the y ’s can be numerical values or class labels, we can develop a predictor ϕ(x,ℒ) that estimates y for an input x. By utilizing several training samples $\left\{{ℒ}_{k}\right\}$ from the same original dataset, our goal is to enhance prediction accuracy compared to using a single dataset φ(x,ℒ) .

For numerical predictions, results are aggregated by averaging predictions from the different samples, expressed as $\phi_{A}(x)=E_{ℒ}\varphi (x,ℒ)$ , where $E_{ℒ}\;$ denotes the expectation over the samples (Breiman, 1996). In the case of class predictions, a voting mechanism is employed, counting how many times each class is predicted across the samples, defined as $N_{c}=\mathrm{nr}\{k;\phi (x,{ℒ}_{k})=c\}$ (Breiman, 1996). The class with the highest count is selected as the final prediction: $\phi_{A}(x)={\text{argmax}}_{c}N_{c}$ (Breiman, 1996). This process, known as “bootstrap aggregating” or bagging, involves generating multiple bootstrap samples $\left\{{ℒ}^{\left(B\right)}\right\}$ from the original dataset, each containing N randomly chosen instances (with replacement) (Breiman, 1996).

### 3.11 Extremely randomized tree (Extra Tree)

The Extra Tree is a tree-based ensemble method, applicable to both regression and classification problems (Geurts et al., 2006), which has two key parameters: the minimum sample size required to split a node and the number of features randomly selected at each node. This ensemble method can be trained on the complete set of original training instances multiple times, allowing for the construction of robust models that enhance predictive performance. The number of trees in this ensemble is denoted by *M*. In classification problems, the final prediction is determined by a majority vote, while in regression problems, it is determined by the arithmetic average of the predictions made by the trees. The rationale for the Extra Trees approach stems from the bias-variance perspective, which posits that rigorous randomization of dividing points and features, together with ensemble averaging, can significantly reduce variance compared to less randomized methods.

In place of bootstrap replicates, the complete original training sample is used to minimize bias. Similar to other tree-growing methods, the computing expense of the procedure is about equal to NlogN when considering the size of the learning sample. N in this instance indicates the total number of data points. NlogN is considered to have a very efficient time complexity. It shows the algorithm’s performance does not decrease much as the dataset grows. To further enhance the model’s performance, the parameters were customized by adjusting the following values: “number of estimators: [100, 200, 300],” “maximum tree depth: [None, 10, 20, 30],” “minimum samples to split: [2, 5, 10]” and “minimum samples per leaf: [1, 2, 4].”

### 3.12 Light Gradient Boosting Machine (LightGBM)

Light Gradient Boosting Machine (LightGBM) is a gradient boosting framework introduced by Ke et al. (2017) to enhance computational efficiency by reducing the number of data instances and features. This model employs two key techniques: gradient-based one-side sampling (GOSS) and exclusive feature bundling (EFB) (Ke et al., 2017). GOSS selectively retains instances with large gradients while sampling from those with small gradients, adjusting information gain with a constant multiplier to mitigate sampling bias (Ke et al., 2017). For example, if a=0.4 , GOSS keeps the top 40% of instances based on gradient values and randomly selects b×100% from the remaining instances. EFB constructs a graph with weighted edges (Ke et al., 2017) to represent feature conflicts, organizing features based on their degrees. Features with minor conflicts are bundled together, improving efficiency by using a ranking strategy based on nonzero counts rather than generating a complete graph (Ke et al., 2017).

To optimize LightGBM’s performance, hyperparameters were adjusted, including the number of boosting iterations, number of leaves, learning rate, and regularization strengths, with settings such as “number of boosting iterations: [100, 200, 300],” “number of leaves: [31, 50, 100],” “learning rate: [0.01, 0.1, 0.2],” “L1 regularization strength: [0, 0.1, 0.5],” “L2 regularization strength: [0, 0.1, 0.5],” “subsample ratio: [0.8, 1.0],” and “feature fraction: [0.8, 1.0].”

### 3.13 Integrating LightGBM with LLM for interpretation of the outcome

**Figure 2:**
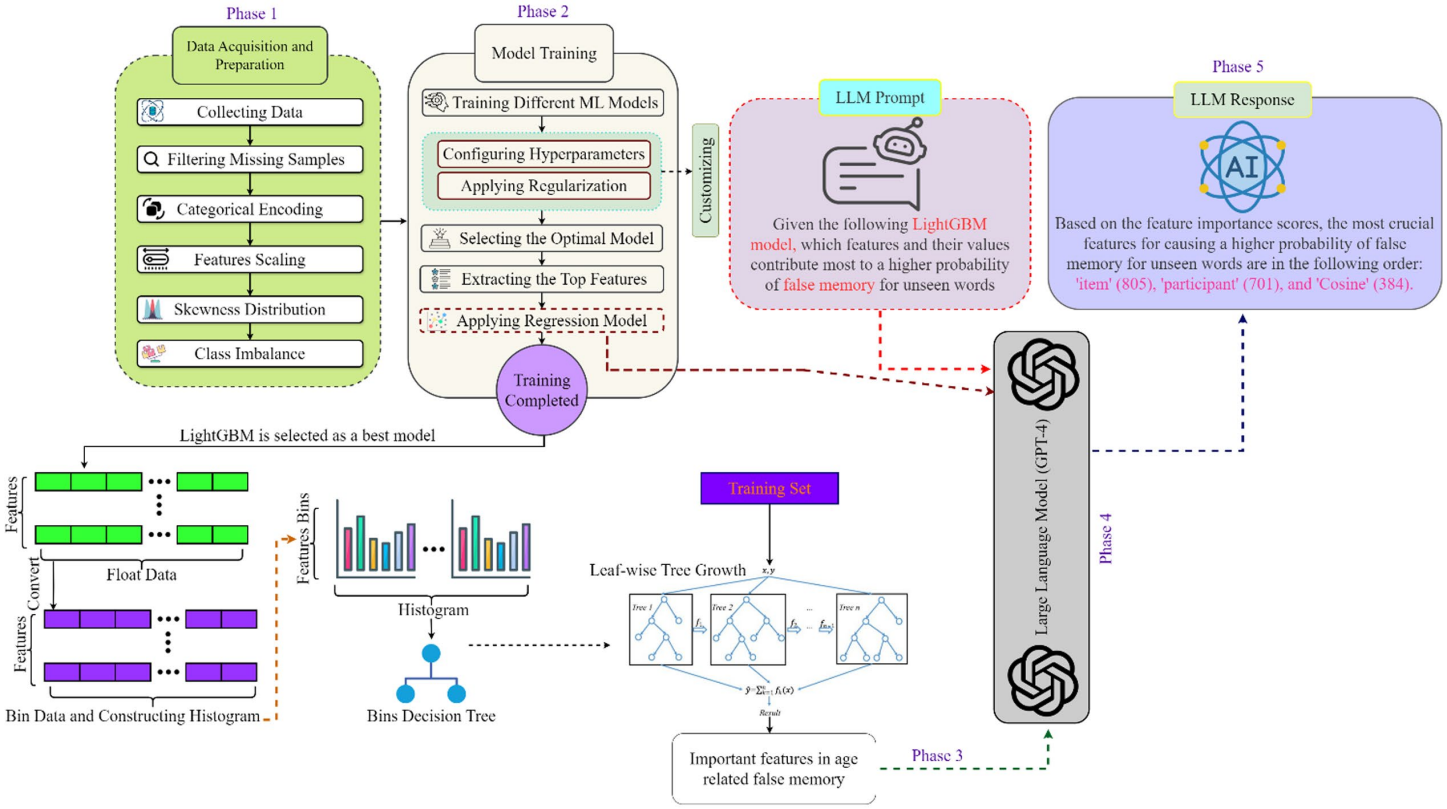
The architecture of this study, the five phases include data acquisition, model training, model prediction, LLM integration, and response generation, moving from dataset preparation to LLM-driven interpretation of model results.

Figure 2 illustrates the five phases of this study’s architecture: data acquisition, model training, prediction result, large language model (LLM) integration, and response generation. The data acquisition phase, which outlines the processes for collecting and preparing a high-quality dataset, is thoroughly explained in Section 3.1 and 3.2. In the second phase, model training, various machine learning techniques were applied to this dataset. After that, some of the model’s hyperparameters were adjusted using regularization strategies, and the outcomes were monitored to maximize performance. The experiments revealed that the modified LightGBM model outperformed other traditional machine learning models; therefore, this model was used as a benchmark, identifying the top features (using the permutation feature importance technique) that produced satisfactory results during training. Finally, a mixed-effects logistic regression model was employed to analyze feature interactions, revealing key trends related to cognitive decline and memory patterns.

In the third phase, prediction result, the modified LightGBM model provides the prediction results, starting with a training dataset. The data is first converted to float format and then used to create histograms, which facilitate the generation of bin decision trees. Subsequently, these decision trees are developed in a leaf-wise manner, meaning that new leaves are added one by one to the tree. The model is trained on the training set, and the predictions of all trees are aggregated to produce the final output, as seen in Figure 2.

During the fourth phase, LLM integration, the LLM (e.g., GPT-4) was employed to detect additional patterns related to memory. Specific prompts were designed to determine which factors most significantly influenced false memory across various age groups. The results from the modified LightGBM and logistic regression models were also input into the LLM. The logistic regression model provided statistical outputs, encompassing coefficients, standard errors, *z*-scores, *p*-values, and confidence intervals. These outputs offered insights into the strength and direction of each variable’s impact, the significance of parameters, the extent of deviation, statistical significance, and the range of parameters. By combining this data with the prompts, the LLM generated detailed explanations about the features that had the greatest impact on false memory (phase 5). Section 4.2.5 provides detailed explanations of the prompts and the responses generated by LLM.

## 4 Experimental results and discussion

### 4.1 Experimental design

This section discusses the dataset used in the study, the data cleaning process, and the evaluation techniques employed to assess the performance of the various models. Specifically, Section 4.1.1 explains the dataset and its characteristics in detail; Section 4.1.2 outlines the steps taken to prepare the dataset; and Section 4.1.3 demonstrates the model validation techniques.

#### 4.1.1 Dataset

A set of 60 experimental sentences was constructed for a self-paced reading task. Each sentence was manipulated to include a target noun (e.g., *bear*) that was either post-modified (e.g., *the bear that was injured and dangerous*), pre-modified (e.g., *the injured and dangerous bear*), or unmodified (i.e., *the bear*). The unmodified sentences were minimally adapted from their modified counterparts so that the overall sentence structure remained consistent across all versions of an experimental sentence. This design choice was made to minimize confounding variables related to sentence length and verb position (i.e., *chased*) in the analysis. A sample experimental sentence is illustrated in Table 1: *It was the injured and dangerous bear [target] that the hunters chased in the dense forest yesterday.*

**Table 1:** Example stimuli from the self-paced reading task.

Condition	Sentence
Post-modified	It was the bear that was injured and dangerous that the hunters chased in the cold forest yesterday.
Post-unmodified	The video footage showed that it was the bear that the hunters chased in the cold forest yesterday.
Pre-modified	It was the injured and dangerous bear that the hunters chased in the cold forest yesterday.
Pre-unmodified	The footage showed it was the bear that the hunters chased in the cold forest yesterday.

There were 20 sentences per condition—post-modified, pre-modified, and unmodified—for each experimental list, accompanied by 100 filler sentences, totaling 160 sentences per list. None of the experimental sentences were repeated in any of the lists. Additionally, comprehension questions were included for 40 experimental sentences and 50 filler sentences to ensure participant attention during the task. A set of 300 experimental items was prepared for a subsequent surprise recognition memory task conducted immediately after the reading phase. During the memory test, participants were presented with single words and asked to make a binary decision regarding whether the words had appeared during the reading task. Each experimental sentence was associated with 5 memory items: two words that were present in the corresponding sentence (e.g., *bear*, *hunters*, see Table 1), and three new items that were not present in any of the experimental or filler sentences but were semantically related to the target word (i.e., *bear*) to varying degrees.

#### 4.1.2 Data preparation

Before analysis, the data were cleaned to ensure quality standards suitable for use in machine learning algorithms. The original dataset contained missing or extreme values that could distort analyses; therefore, such data needed to be filtered to maximize quality. Multiple cleaning procedures were applied, including categorical encoding, imputing missing values, mitigating skewed distributions, detecting outliers, feature scaling, and addressing class imbalance. Regarding categorical encoding, it is recommended to use vector or numerical data when implementing learning algorithms. To accomplish this, One-Hot Encoding was used (Potdar et al., 2017), which assigns numerical labels to categorical data.

To further preserve the semantic content in the dataset, missing records were filled with mean values rather than being removed entirely. A dataset distribution is considered skewed or asymmetric if its left and right sides are not evenly distributed around the mean (Sedgwick, 2012). To evaluate distribution symmetry, techniques such as box plot visualization, histograms, and quantile-quantile (Q–Q) plots were used. The dataset was found to exhibit skewness, and the Box–Cox method (Box and Cox, 1964) was applied to reduce this issue. Additionally, *z*-score and interquartile range (IQR) techniques were used to identify potential extreme values (i.e., outliers). To improve data quality, the Isolation Forest approach (Liu et al., 2008) was used with a 1% contamination rate to detect and eliminate probable outliers. Rather than replacing outliers with alternative values (e.g., maximum valid value for a participant), these values were entirely removed from the dataset.

Feature scaling was performed using the RobustScaler technique, which keeps the values within a consistent range. By scaling data based on the interquartile range and centering it on the median, RobustScaler minimizes the impact of outliers (de Amorim et al., 2023). Finally, the dataset exhibited class imbalance, where each class did not contain equal amounts of data. To address this, the Synthetic Minority Oversampling Technique (SMOTE) library was utilized (Chawla et al., 2002), which generates synthetic data samples and effectively mitigates class imbalance issues.

#### 4.1.3 Evaluation metrics

To further analyze the performance of different machine learning models, several validation techniques were applied, including the confusion matrix (Marom et al., 2010), receiver-operating characteristic curve (ROC), area under the curve (AUC) (Verbakel et al., 2020), and k-fold cross-validation (Wong and Yeh, 2019). Confusion matrices were used because model performance cannot be determined from a single classification report alone. Since the confusion matrix provides detailed information about total prediction outcomes, it serves as a suitable method for evaluating model results. In addition, the ROC-AUC curve offers insight into how accurately a binary classification model can separate data points into positive and negative classes. Cross-validation techniques were also employed to minimize overfitting and reduce model bias. This method introduces variation into the training pipeline, providing a more comprehensive understanding of model performance. These validation methods are discussed in detail in Section 4.2.2.

### 4.2 Results and discussion

This section presents a detailed discussion of the experimental results and insights derived from various ML and DL approaches. Section 4.2.1 explains the classification reports of several ML models tested in the study. Section 4.2.2 describes the validation techniques used to evaluate the performance of ML models. Section 4.2.3 demonstrates the importance of feature and interaction modeling. Section 4.2.4 provides a comparative analysis of different DL models. Section 4.2.5 illustrates the interpretation process involving the use of LLMs. Finally, Section 4.2.6 presents a qualitative comparative analysis of existing studies.

#### 4.2.1 Classification report summary on multiple models

Table 2 provides information on different ML models and their classification reports, such as precision, recall, and F1-scores on binary classification (0 and 1) tasks. In this classification report, twelve different ML models were used, including Naive Bayes (Huang and Li, 2011), AdaBoost (Freund and Schapire, 1997), Support Vector Machine (SVM) (Boswell, 2002), Decision Tree (Lu et al., 2022), K-Nearest Neighbors (KNN) (Cunningham and Delany, 2020), Voting Classifier (Breiman, 1996), Random Forest (Breiman, 2001), XGBoost (Chen and Guestrin, 2016), Bagging (Breiman, 1996), CatBoost (Dorogush et al., 2018), Gradient Boosting (Friedman, 2001), and LightGBM (Ke et al., 2017).

**Table 2:** Classification performance of different models.

Model	Class 0			Class 1		
	Precision	Recall	F1	Precision	Recall	F1
Naïve Bayes	0.55	0.44	0.49	0.54	0.64	0.59
Modified AdaBoost	0.63	0.63	0.63	0.64	0.63	0.63
SVM	0.62	0.70	0.66	0.66	0.58	0.62
Modified decision tree	0.69	0.70	0.69	0.70	0.68	0.69
Modified KNN	0.68	**0.87**	0.76	0.82	0.59	0.69
Voting Classifier	0.74	0.77	0.75	0.76	0.74	0.75
Modified random forest	0.77	0.78	**0.78**	0.78	0.77	0.78
Modified XGBoost	0.80	0.74	0.77	0.77	0.82	0.79
Bagging	0.86	0.71	**0.78**	0.76	**0.88**	0.82
CatBoost	**0.87**	0.69	0.77	0.75	**0.90**	0.82
Modified gradient boosting	0.85	0.72	**0.78**	0.76	**0.88**	0.88
Modified LightGBM	0.84	0.73	**0.78**	0.76	0.87	0.81

Bold values indicate the highest performance scores for each metric across all models.

Table 2 illustrates that only the modified LightGBM model outperforms the other machine learning models. Other approaches, such as CatBoost and Bagging, exhibit slight variations in their precision, recall, and F1-score metrics. Notably, Bagging demonstrates a high recall and F1-score for class 0, whereas CatBoost achieves a high precision for the same class. In Class 1, Bagging exhibits higher precision than CatBoost; however, its recall value is lower. The Random Forest model demonstrates ideal performance across precision, recall, and F1-score, with both classes performing well. The results from the Voting Classifier show minimal significance, as reflected in Table 2. Additionally, KNN and AdaBoost do not produce satisfactory results. On the other hand, LightGBM and Gradient Boosting show minimal difference in recall. More specifically, LightGBM shows an improved recall of 73% for class 0 compared to Gradient Boosting, which yielded 72%. Finally, the Naive Bayes model demonstrated the weakest performance among all methods used to classify responses in the psychology dataset.

#### 4.2.2 Model assessment and validation

**Figure 3:**
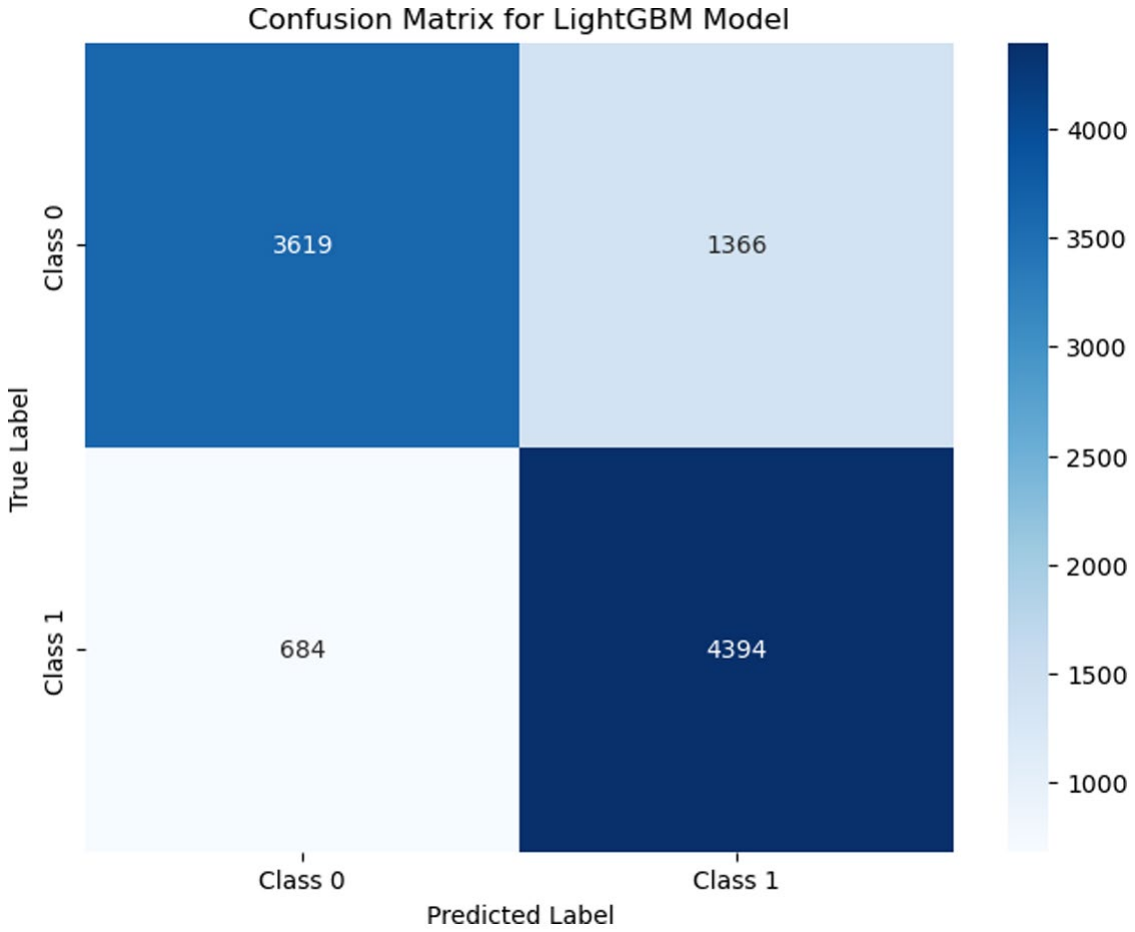
Visualizing confusion matrix for the LightGBM model in binary classification, displaying the true positives, false positives, true negatives, and false negatives.

This section describes the model evaluation techniques that were considered to measure the performance of several ML models. Firstly, a confusion matrix was used to evaluate the performance of different learning algorithms. It can be applied to both binary and multiclass classification tasks (Heydarian et al., 2022). The confusion matrix is formed based on four values: true positive (TP), false positive (FP), true negative (TN), and false negative (FN). In Figure 3, a total of 3,619 samples were accurately predicted as class 0 (TN), and 4,394 were recorded as class 1 (TP). The total FP count was 1,366, representing instances where class 0 was predicted as class 1. Finally, the FN value was 684, indicating instances that were expected to be class 1 but were predicted as class 0.

**Figure 4:**
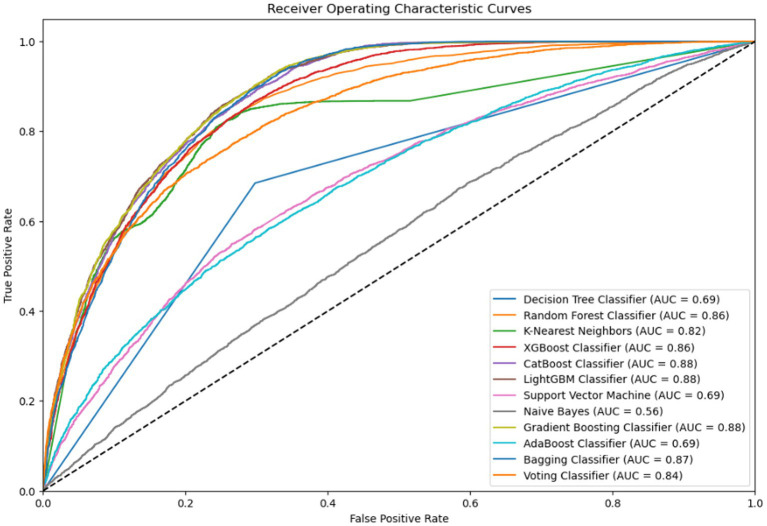
The models’ total performance is shown by the ROC-AUC curve, which also illustrates how well it can differentiate between positive and negative classes.

Subsequently, 5-fold cross-validation was applied to improve the performance stability of the modified LightGBM model. In addition, the ROC-AUC technique, shown in Figure 4, was used to further evaluate model performance. The ROC curve is constructed using the true positive rate (TPR) and false positive rate (FPR), while AUC provides a probability-based score to assess the overall classification capability. AUC values typically range from 0 to 1, with higher values indicating better model performance. According to Figure 4, the LightGBM model achieved an AUC of 0.88, indicating strong capability in distinguishing between classes 0 and 1.

#### 4.2.3 Feature importance and interaction modeling

In this section, the importance of features extracted using the modified LightGBM model with interaction modeling is discussed. The permutation feature importance technique was used to identify essential features of the predictive model. The primary goal of this technique is to understand how model scores decrease when a single feature value is randomized (Galli, 2024). This technique provides an intuitive grasp of the function each feature plays in the model’s final conclusion, allowing for greater insight into what information the model relies on the most when making predictions.

**Figure 5:**
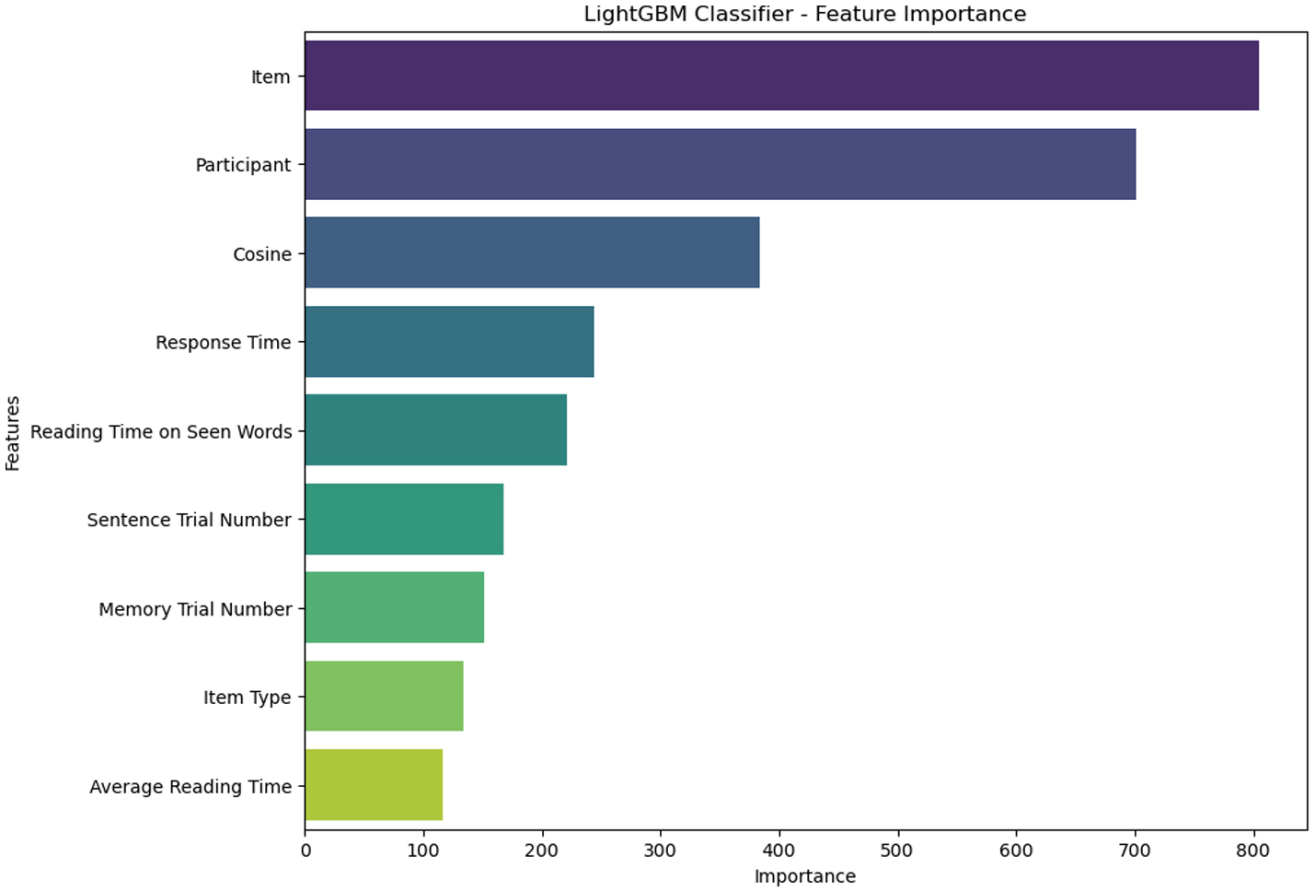
Demonstrating the significant features extracted using the LightGBM model.

Figure 5 displays the 9 most important features extracted from the LightGBM model. These features include “item” (i.e., the single memory words/items in the surprise recognition memory test), “participant,” “Cosine” (which quantifies the semantic similarity between the target word (i.e., bear), and the memory item), “Response time” (i.e., the time it took for a participant to make a decision about the current memory item), “Reading Time on Seen Words” (i.e., the average reading/encoding time on the two memory items that were present in the critical sentences), “Sentence Trial Number” (i.e., the linear position of the sentence associated with the current memory item which ranged from 1 to 160), “Memory Trial Number” (i.e., the linear position of the memory items, ranging from 1 to 300), “Item Type” (i.e., whether the memory item was present in any of the sentences or not, resulting in Seen and Unseen types), and “Average Reading Time” (i.e., the time spent reading the entire sentence corresponding to the memory item). These variables capture both behavioral (e.g., response time, reading time) and semantic (e.g., cosine similarity) aspects of memory performance, demonstrating the cognitive complexity of the recognition task.

As shown in Figure 5, the “item” is the most important feature, with a score of 805. “Participant” is in the second position with a score of 701. The “Cosine” feature is the third. “Response Time” is placed in the fourth position with a value of 244. “Reading Time on Seen Words” is in the fifth position with a value of 221. “Sentence Trial Number” is ranked sixth with a score of 168, while “Memory Trial Number” is ranked seventh. The remaining two features, “Item Type” and “Average Reading Time,” are ranked eighth and ninth, with scores of 134 and 117, respectively. The ranking of these features indicates that the model’s predictions were substantially influenced by both content-specific variables (e.g., semantic similarity and item identity) and individual-level or contextual variables (e.g., participant, trial positions).

**Figure 6:**
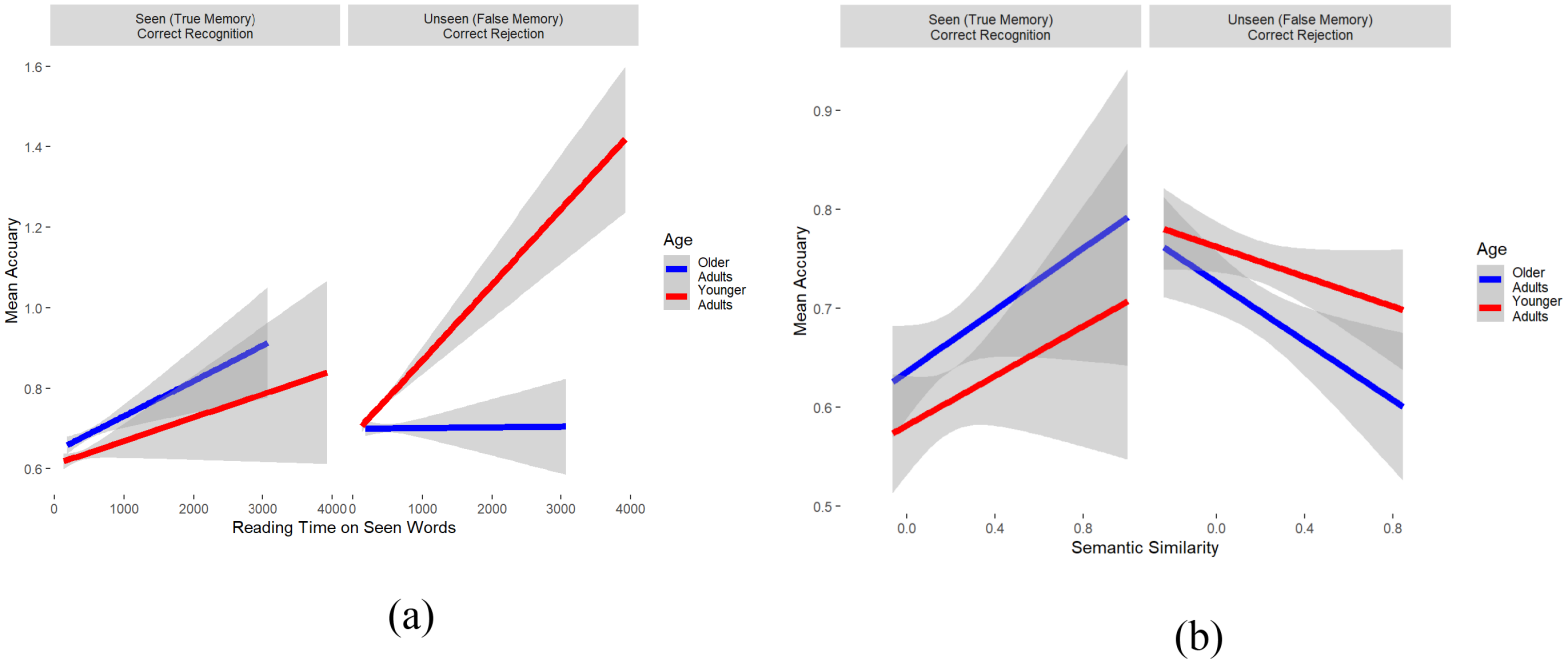
Comparison of reading time and semantic similarity with mean accuracy across different age groups and item types. **(a)** Relationship between reading time on seen words and mean accuracy for correct recognition (true memory) and correct rejection (false memory) across age groups. **(b)** Relationship between semantic similarity and mean accuracy for correct recognition (true memory) and correct rejection (false memory) across age groups.

To corroborate the effects of these features, and more importantly, whether and how they interact with Age, a logistic mixed-effects regression model was conducted predicting accuracy as a function of the five extracted features, as well as all their two-way and three-way interactions with Age and Item Type (Seen vs. Unseen). Two main results were observed: First, as shown in Figure 6a, longer reading times on seen words (i.e., *bear* and *hunters*) increased their correct recognition (true memory) for both younger and older adults (left panel). However, although longer reading times on the seen words increased correct rejection of lures for younger adults (Est. = 0.075, SE = 0.05, *z* = 1.37, *p* = 0.17), it did not have any effect on older adults. In fact, it slightly increased false memory for older adults (Est. = −0.018, SE = 0.04, *z* = −0.44; *p* = 0.66, right panel). Second, as shown in Figure 6b, greater semantic similarity (between the target word and the lures) increased correct recognition of seen words for both groups (left panel) and decreased false memory for lures in both groups (right panel). However, the decrease in accuracy for unseen words as a function of semantic similarity was relatively greater for older adults (Est. = −0.26, SE = 0.07, *z* = −3.38, *p* < 0.001) than for younger adults (Est. = −0.16, SE = 0.07, *z* = −2.43, *p* = 0.01). In other words, with increasing semantic similarity (operationalized by cosine), false memory increased more for older than younger adults (right panel). These interactions strengthen the model’s dependence on cognitively significant traits and provide interpretable avenues for comprehending age-related differences in memory performance and false recognition.

#### 4.2.4 Quantitative comparison with deep learning models

Table 3 presents the classification report of the deep learning models and compares them with the optimized LightGBM model (Ke et al., 2017). Given the satisfactory outcome of the optimized ML model, several deep learning and transformer models were tested, including Convolutional Neural Network (CNN) (LeCun et al., 2010), Long Short-Term Memory (LSTM) (Hochreiter, 1997), Bidirectional Long Short-Term Memory (BI-LSTM) (Graves et al., 2005), Recurrent Neural Network (RNN) (Rumelhart et al., 1986), Stack-LSTM (Wang et al., 2018), Stack-BI-LSTM (Ran et al., 2020), Transformer (Waswani et al., 2017), and TabNet (Arik and Pfister, 2021). These models were explored to determine whether they could capture semantic patterns from the dataset. According to Table 3, the traditional transformer model performed the worst. This indicates that although the transformer architecture performs well on other downstream tasks, it is not as effective for this dataset where capturing meaningful patterns is crucial. Similarly, TabNet—a transformer-based model developed by researchers at Google Cloud for tabular data—also showed suboptimal performance. Among the deep learning models evaluated, Stack-BI-LSTM showed relatively better results. Nevertheless, the modified LightGBM model outperformed all other models examined in this study.

**Table 3:** Classification performance of deep learning based algorithms.

Model	Class 0			Class 1		
	Precision	Recall	F1	Precision	Recall	F1
Transformer	0.50	**1.00**	0.66	0.00	0.00	0.00
TabNet	0.56	0.56	0.56	0.56	0.56	0.56
RNN	0.59	0.66	0.62	0.61	0.53	0.57
CNN	0.65	0.72	0.68	0.68	0.60	0.64
LSTM	0.64	0.67	0.65	0.65	0.61	0.63
Stack-LSTM	0.68	0.71	0.69	0.69	0.67	0.68
BI-LSTM	0.68	0.76	0.72	0.72	0.64	0.68
Stack-BI-LSTM	0.72	0.80	0.76	**0.77**	0.69	0.73
Modified LightBM	**0.85**	0.73	**0.78**	0.76	**0.88**	**0.82**

Bold values indicate the highest performance scores for each metric across all models.

#### 4.2.5 Interpretation using LLM

**Figure 7:**
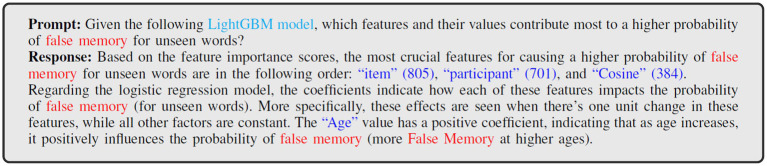
Analysis of prompts with corresponding inputs and outputs: features contributing to false memory probabilities for unseen words.

To further analyze the patterns and understand hidden insights, we used prompt engineering techniques based on the GPT-4 LLM. The goal of this prompt integration was to examine how each feature interacts with the others and to gain a better understanding of cognitive decline patterns, such as false memory for unseen words. Figure 7 shows a prompt in which a human-written inquiry is sent into the model, and the model provides an output as a response. We initially fed the key elements that contribute to a greater likelihood during modified LightGBM training to the LLM. Once the important information was obtained, the LLM began integrating model interpretations using insights from the logistic mixed-effects regression model, focusing on how age interacts with other characteristics to influence memory performance. Then, the LLM responded with a narrative that explains the most significant aspects that have the best possibility of identifying a false memory pattern.

#### 4.2.6 Qualitative comparative analysis of existing studies

Table 4 gives information about different ML and DL models that were used on various data modalities, such as numeric, DNA, image, speech, and longitudinal data. As shown in the table, it is evident that some of the studies included participants between the ages of 40 and 90, aiming to understand cognitive decline and associated patterns. It can also be observed that most studies focusing on ML and DL techniques remain underutilized, suggesting that traditional ML models may still perform effectively in this domain. In terms of model validation, it was found that several recent studies did not validate their proposed ML models for detecting cognitive decline. Furthermore, it is noteworthy that LLMs have not been used in previous research, indicating significant potential for their application in the field of psychology. Although some studies applied ensemble techniques such as Random Forest, none utilized boosting methods like Gradient Boosting or LightGBM. Given the strong performance of boosting techniques in other healthcare-related downstream tasks (Ganie et al., 2023; Yang et al., 2023; Fourkiotis and Tsadiras, 2024), exploring these approaches in cognitive decline and false memory pattern recognition appears promising.

**Table 4:** Summary of models detecting cognitive decline.

Study	Data type	Age range	Age compared	ML	DL	Validation	Best model	LLM
Revathi et al. (2022)	Numeric	40–65	✓	✓	✖	✖	Multinomial logistic	✖
Casanova et al. (2020)	DNA	65–90	✓	✓	✖	✖	Random Forest	✖
Almgren et al. (2023)	Longitudinal	✖	✖	✓	✖	✖	✖	✖
Hosseinzadeh et al. (2023)	SPECT	✖	✖	✖	✓	✓	MLP	✖
Chandler et al. (2023)	Speech	✖	✓	✓	✖	✖	Unimodal ML	✖
Our model	Numeric	18–85	✓	✓	✓	✓	Modified LightGBM	✓

On the other hand, the studies listed in Table 4 did not consider younger age groups when detecting cognitive decline. While false memory is more commonly observed in older populations, there may be contexts in which younger individuals also exhibit memory limitations. Therefore, a comprehensive comparison across age groups is essential to gain a fuller understanding of memory behavior. The present study addresses these gaps by comparing younger and older adults and extracting meaningful insights through analysis. For instance, instances were identified where older adults demonstrated better memory performance than their younger counterparts. With respect to ML, the LightGBM model was used and achieved optimal results through systematic evaluation. Additionally, various DL and transformer architectures were applied to detect significant patterns, although their performance in identifying cognitive decline and false memory patterns was found to be suboptimal. LLMs were also incorporated and combined with prompt engineering to further identify memory-related patterns. This qualitative comparison is expected to guide future research efforts focused on age-related false memory.

## 5 Conclusion

Aging is associated with cognitive decline in crucial functions such as language processing, memory, and attention which can affect the quality of life in older adults. Although there are negative consequences caused by this cognitive decline, little is known about the mechanisms of false memory. In this study, optimized machine learning algorithms combined with permutation importance and LLM were used to isolate the most important features affecting true and false memory in younger and older adults following a reading experiment. The potential interactions of the identified features with Age were then examined. The results revealed greater vulnerability of older adults to false memory (i.e., claiming to have seen words that were absent during reading), especially when the memory items were semantically more similar to the target words. This observation lends support to the Inhibition Deficit Hypothesis because it clearly shows that older adults are less capable of suppressing semantically similar words during memory retrieval. An inhibition deficit may lead to broader and stronger spreading activation from the target words to semantic neighbors for older relative to younger adults, boosting the memory activation for the semantic neighbors and resulting in greater false memory rates for older adults. In addition, unlike younger adults, older adults did not benefit from longer encoding time on seen words to correctly reject unseen memory items, which suggests that older adults may not be able to encode information as robustly as younger adults, compromising their memory performance. Taken together, these results show that encoding time and similarity-based interference are two factors influencing false memory and highlight the importance of machine learning as a tool for uncovering the behavioral patterns underlying age-related cognitive decline.

Nevertheless, this study is characterized by numerous constraints. Although the dataset utilized is informative, it may not encompass the complete spectrum of contextual or semantic influences that are present in real-world memory scenarios. Furthermore, the generalizability of the results may be limited by the task specificity and the absence of multimodal or longitudinal data.

Future research should investigate the applicability of these findings by examining memory performance in various cognitive contexts and by utilizing a broader, more diverse set of datasets. Additionally, the interpretability of the model could be improved by incorporating real-time behavioral monitoring and neurocognitive signals.

## Data Availability

The datasets presented in this article are not readily available because the dataset used in this study contains cognitive and memory performance data from younger and older adults. Due to ethical considerations and privacy regulations, access to the dataset is restricted. The data includes sensitive participant information. Therefore, sharing of the dataset is limited to approved research collaborations and is subject to appropriate data-sharing agreements. Requests to access the datasets should be directed to hk702@msstate.edu.
